# Endocrine disrupting chemicals interfere with decidualization of human primary endometrial stromal cells *in vitro*

**DOI:** 10.3389/fendo.2022.903505

**Published:** 2022-08-19

**Authors:** Darja Lavogina, Nadja Visser, Külli Samuel, Eva Davey, Richelle D. Björvang, Jasmin Hassan, Jani Koponen, Panu Rantakokko, Hannu Kiviranta, Ago Rinken, Matts Olovsson, Andres Salumets, Pauliina Damdimopoulou

**Affiliations:** ^1^ Institute of Chemistry, University of Tartu, Tartu, Estonia; ^2^ Competence Centre on Health Technologies, Tartu, Estonia; ^3^ Department of Women´s and Children’s Health, Uppsala University, Uppsala, Sweden; ^4^ Division of Obstetrics and Gynecology, Department of Clinical Science, Intervention and Technology, Karolinska Institutet and Karolinska University Hospital, Stockholm, Sweden; ^5^ Department of Health Security, Finnish Institute for Health and Welfare, Kuopio, Finland; ^6^ Department of Obstetrics and Gynecology, Institute of Clinical Medicine, University of Tartu, Tartu, Estonia

**Keywords:** endocrine disrupting chemical, phthalate, persistent organic pollutants (POPs), human endometrium, decidualization, per- and polyfluorinated alkyl substances (PFASs), primary stromal cells, infertility

## Abstract

Multiple studies have shown associations between exposure to endocrine disrupting chemicals (EDCs) and reduced fertility in women. However, little is known about the target organs of chemical disruption of female fertility. Here, we focus on the hormone-sensitive uterine lining, the endometrium, as a potential target. Decidualization is the morphological and functional change that endometrial stromal cells undergo to support endometrial receptivity, which is crucial for successful implantation, placentation, and pregnancy. We investigated the effect of nine selected EDCs on primary human endometrial stromal cell decidualization *in vitro.* The cells were exposed to a decidualization-inducing mixture in the presence or absence of 1 μM of nine different EDCs for nine days. Extent of decidualization was assessed by measuring the activity of cAMP dependent protein kinase, Rho-associated coiled-coil containing protein kinase, and protein kinase B in lysates using photoluminescent probes, and secretion of prolactin into the media by using ELISA. Decidualization-inducing mixture upregulated activity of protein kinases and prolactin secretion in cells derived from all women. Of the tested chemicals, dichlorodiphenyldichloroethylene (p,p’-DDE), hexachlorobenzene (HCB) and perfluorooctanesulfonic acid (PFOS) significantly reduced decidualization as judged by the kinase markers and prolactin secretion. In addition, bisphenol A (BPA) reduced prolactin secretion but did not significantly affect activity of the kinases. None of the EDCs was cytotoxic, based on the assessment of total protein content or activity of the viability marker casein kinase 2 in lysates. These results indicate that EDCs commonly present in the blood circulation of reproductive-aged women can reduce decidualization of human endometrial stromal cells *in vitro*. Future studies should focus on detailed hazard assessment to define possible risks of EDC exposure to endometrial dysfunction and implantation failure in women.

## Introduction

The environment is polluted by a wide range of human-made industrial chemicals, which are deliberately or accidently released by humans (1). Some of them can interfere with the endocrine system leading to adverse health effects in humans and wildlife, and can be termed as endocrine disrupting chemicals (EDCs) according to commonly accepted definitions (2). The mechanisms of endocrine disruption have diversified from the classical EATS modalities (Estrogen, Androgen, Thyroid and Steroidogenesis) to encompass *e.g.* signal transduction, epigenetics and cell fate in hormone-producing or hormone-sensitive cells (3). EDCs can be found in various natural environments including water, soil, and air (4). Many consumer products such as food packaging, kitchen utensils, plastics, personal care products, home electronics, textiles, and furniture contain EDCs (5, 6). Therefore, humans and wildlife are continuously exposed to mixtures of possibly hazardous contaminants.

Despite extensive and continuous exposure documented in multiple humanbiomonitoring studies (*e.g*. National Biomonitoring Program by the Centers for Disease Control and Prevention in the US, and the HBM4UE in the European Union), hazard identification and characterization of most chemicals on the market remains limited. For example, out of the 100 000 chemicals on the European market, only 500 have been thoroughly characterized for their hazards and exposures (7). In addition, typical guideline assays for reproductive toxicity are not well suited for detecting fertility disrupting effects of chemicals in women, which has led to the European Commission flagging female reproductive toxicity as a priority research area (8). More research is needed to better understand the effects of EDCs on reproductive health in women, and to develop novel predictive and human-relevant assays that can be used to characterize reproductive hazards of chemicals.

The female reproductive system has multiple essential functions that enable fertility. Ovaries produce competent oocytes that can be fertilized by sperm in the fallopian tubes. The fertilized embryos then travel to the uterus where they implant into the endometrium for further development. Embryo implantation is only possible during the short period of endometrial receptivity, referred as the window of implantation when the epithelial cells have obtained an adhesive phenotype and stromal cells have decidualized. Decidualization is a morphological and functional change of endometrial stromal fibroblasts in the early secretory phase to epithelial-like decidual stromal cells in the mid-secretory phase. Rising levels of the steroid hormones estradiol and progesterone from ovaries together with increased intracellular cyclic adenosine monophosphate (cAMP) levels activate a crosstalk of different downstream gene transcription and protein kinases such as cAMP-dependent protein kinase (PKA) - among other outcomes, resulting in secretion of prolactin (PRL) and insulin growth factor-binding protein 1 (IGFBP-1), classical markers of decidualization (9, 10). Subsequent embryo implantation relies on tight and timely communication between the embryo and the endometrium, which paves the way to controlled trophoblast invasion into the stromal tissue. Decidualized stromal cells are more supportive for trophoblast expansion than undifferentiated stromal cells (11) and decidualization is required to distinguish poor and good quality embryos, favoring the continuation of the pregnancy resulting from the latter (12).

Approximately 15% of known pregnancies end in miscarriage in humans, and this number is likely higher because the very early miscarriages often go unnoticed (13). Miscarriages contribute to longer time-to-pregnancy (14) and are associated with increased risk for future miscarriages or recurrent pregnancy loss (15) and infertility - the inability to achieve pregnancy after 12 months of regular unprotected sexual intercourse (16). Many epidemiological studies have found significant associations between exposure to EDCs, miscarriage and pregnancy complications such as preeclampsia, preterm birth, and fetal growth restriction (17–20). As decidualization is a strictly hormone-dependent, key process in establishment of a pregnancy, and disrupted implantation and placentation can lead to complications mentioned, it is essential to investigate the effects of EDCs on the human endometrium. For example, EDCs affect steroid hormone receptors, affecting endometrial stromal cell signalling pathways and proliferation *in vivo* in rodents (21, 22). Importantly, there is a need to study EDC effects on decidualization in human-relevant settings because there are major differences in endometrial biology between experimental animals like mice and humans (23, 24). Effects of EDCs on human endometrial stromal cells can be studied in controlled settings for example using *in vitro* models where decidualization is triggered with hormones in combination with the cell-permeable analogues of cAMP.

Up to date, only a few studies have investigated the effects of EDCs on human *in vitro* decidualization (**Table 1**) (25–33). Six out of nine published studies have focused on the plastic additive BPA and found that BPA exposure has significant effects on markers of decidualization such as PRL, although results were inconsistent. Some studies found that BPA reduced PRL at higher (87.6 µM) (32) and others at lower (1 µM) (31) concentrations, while some found no effects (28), and others suggested that BPA might even increase PRL secretion (25). This lack of consistency may depend on different decidualization protocols, exposure times and concentrations of EDCs used, and source of cells. Cells for *in vitro* decidualization are often derived from hysterectomy material or endometrial biopsies at different stages of the menstrual cycle, but even commercially available cell lines (THESC) have been used. Patient-derived primary stromal cells typically show high inter-individual variation while commercial cell lines are more homogeneous. However, immortalized commercial cell lines might not reflect true endometrial biology. All these factors complicate the comparison of results between different studies (**Table 1**). In addition to BPA, PFOS, alkylphenols, and triclosan have been found to affect decidualization markers *in vitro* (**Table 1**). Collectively, these nine studies suggest that *in vitro* decidualization is sensitive to EDC exposures.

**Table 1:** Summary of literature on the effects of EDCs on human endometrial stromal cell decidualization *in vitro*.

Origin of cells	EDC exposure (concentrations, time)	Decidualization protocol	Key results	Reference
Endometrial biopsies from gynecological surgery, proliferative state (n=8)	BPA and TCL 100 µM, 100 nM, 100 pM, 24 h, 48 h and 72 h	10 µM P4 for 10 days	TCL and BPA 100 nM and 100 pM arrested cell cycle. Increased expression of decidualization markers *PRL* and *IGFBP1* after 48 h and 72 h. Effects on cell migration genes at 100 µM and 100 nM. Both affected cell migration and PRL and IGFBP-1 secretion at 100 nM.	(25)
First trimester (6–8 weeks) decidual tissue from induced abortion (n=3-5)	PFOS first range from 0.0001 to 1 μM, then 0.01 μM was used 24 h or 48 h	0.5 mM 8-Br-cAMP and 1 µM MPA for 24 h or 48 h	PFOS decreased decidualization markers in a concentration-dependent-way. PFOS attenuates cortisol-induced *PRL* and *IGFBP1* increase. PFOS attenuates cortisone-induced anti-inflammatory response.	(26)
Telomerase-immortalized human endometrial stromal cells (THESC, ATCC CRL-4003) (n=3)	OP and NP 5, 10, 15, 20, 25, or 30 μM. Then 5 μM and 10 μM OP, and 5 μM NP were used 12 days	0.5 mM db-cAMP and 1 µM MPA for 12 days	OP (from 20 µM) and NP (from 15 µM) reduced cell viability, but not for lower concentrations. Decrease in *LEFTY2* and *FOXO1* by OP and NP. PRL and IGFBP-1 expression decreased at day 1 of decidualization with 10 µM OP and 5 µM NP. Effects on secreted decidualization markers at later days.	(27)
Endometrial biopsies from women receiving infertility treatments (n=15)	1 nM to 10 μM BPA, BPF, and BPS 9 days	0.5 mM 8-Br-cAMP, 10 nM E2 and 1 μM P4 for 9 days	No effect on PRL and IGFBP-1 expression. 10 μM BPA or 10 nM and 10 μM BPF, not BPS, increased spheroid invasion area in indirect co-culture. 10 μM BPA or BPF, but not BPS increased spheroid outgrow in direct co-culture. 10 μM BPA increased *LIF* expression and 10 μM BPA and BPF affected anti-invasion molecules.	(28)
Endometrial biopsies from hysterectomies early state cervical cancer, proliferative state and HESC (cell line) (n=6)	BPA 10 pM, 100 pM, 1 nM, 10 nM, 100 nM, 1 μM, 10 μM. 10 μM to study ER 48 h or 6 days	1 μM MPA, and 0.5 mM cAMP for 6 days	Morphological change impaired by 10 nM and 10 μM BPA. Dose-dependent decrease in *PRL*, *IGFBP1* and *HOXA10* mRNA expression. BPA affects methyltransferases dose dependently. BPA decreases histone methylation of *HOXA10, PRL* and *IGFBP-1* promoter region. BPA down regulates MLL1 protein expression and increased EZH2 protein expression through ER.	(29)
Endometrial biopsies from hysterectomy, benign reason (n=8)	BPA 5, 25, 50 and 100 μM 48 h	0.5 mM 8-Br-cAMP 96 h	5 μM and 100 μM BPA decreased proliferation. High doses affect mRNA and protein expression of IGFBP-1 and mRNA of steroid enzymes.	(30)
Endometrial biopsies from hysterectomy or hysteroscopy, proliferative state (n=6)	BPA (1 pM - 1 µM) 24 h	3 days E2 100 nM, then E2+P4 100 nM and 10 µM for 12 days	No effect on proliferation. Reduction of PRL secretion at 1 µM. No effect on IGFBP-1 secretion. Secretion of MIF stimulated. Effect on mRNA/protein levels of different hormone receptors.	(31)
Decidua parietalis of the placental membrane (n=4)	BPA 1 ng/mL to 20 µg/mL 8 days	E2 36 nM, MPA 1 μM and db-cAMP 0.5 mM for 8 days	20 µg reduces *PRL* mRNA expression. 20 µg reduces cell proliferation. 20 µg reduces *ESR1* and *PGR* expression. 20 µg reduces cell cycle gene *CCND2* expression.	(32)
Endometrial biopsies from patients receiving IVF treatment (n=4)	Mancozeb 3 μg/mL 9 days	0.5 mM cAMP, 10 nM 17β-E2 and 1 μM P4 for 9 days	No effect on *PRL* and *IGFBP1* gene expression. Effect on morphology change.	(33)

BPA, Bisphenol A; BPF, Bisphenol F; BPS, Bisphenol S; cAMP, Cyclic adenosine monophosphate; CCND2, Cyclin D2; E2, Estrogen; ER/ESR1, Estrogen receptor alpha; EZH2, Enhancer of Zeste Homolog 2; FOXO1, Forkhead Box Protein O1; HOXA10, Homeobox A10; IGFBP-1/IGFBP1, Insulin-like Growth Factor Binding Protein 1; IVF, In Vitro Fertilisation; LEFTY2, Left-Right Determination Factor 2; LIF, Leukemia Inhibitor Factor; MIF, Macrophage Migration Inhibitory Factor; MLL1, Mixed-Lineage Leukemia 1; MPA, Medroxyprogesterone acetate; NP, Nonylphenol; OP, Octylphenol; P4, Progesterone; PFOS, Perfluorooctane sulfonic acid; PGR, Progesterone Receptor; PRL/PRL, prolactin; TCL, Triclosan.

Here, our ambition was to study the impact of a wide selection of EDCs on human endometrial cell decidualization using our recently established *in vitro* model for decidualization (34). This model is based on primary endometrial stromal cells from hysterectomies (or endometrial biopsies) that are simulated to decidualize with a mixture containing progesterone and estradiol; the process is monitored using a suite of kinases reflecting activation of intracellular signaling, and secretion of PRL as the final outcome (34). In this way, instead of focusing on one marker, we assessed a characteristic fingerprint involving several previously identified markers of decidualization, which is a complex physiological process that cannot be explained by modulation of a single pathway. Apart from assessing PRL secretion and activity of PKA, we explored activity of Rho-dependent protein kinase (ROCK), protein kinase B (Akt/PKB), casein kinase 2 (CK2) and total protein concentration in lysates. The nine EDCs were chosen for the following reasons: BPA and its replacement chemical bisphenol F (BPF) because they have been studied in similar models before (**Table 1**) and BPA is a known human endocrine disrupter (European Chemicals Agency); phthalate MEHP (metabolite of DEHP) because it is also a known human endocrine disrupter (European Chemicals Agency) and associated to miscarriage in humans (18, 20); organochlorine pesticides p,p’-DDE (metabolite of DDT) and HCB, and the polychlorinated biphenyls PCB180 and PCB170, because they associate to longer time-to-pregnancy in our cohort studies (35, 36); HCB is also associated with lower odds for clinical pregnancy and live birth (36); and PFOS and perfluorooctanoic acid (PFOA) because they are associated to preterm birth, fetal growth restriction and pre-eclampsia in humans (19). In addition, the chosen EDCs have different physiochemical properties. BPA, BPF and MEHP are quickly metabolized and excreted from the body; PFOS and PFOA are resistant to degradation (*i.e*., are persistent), amphiphilic and predominantly bind to plasma proteins and accumulate in liver, while p,p’-DDE, HCB, and PCBs are persistent, lipophilic and accumulate in adipose tissue. A concentration of 1 µM was chosen for all EDCs to allow identification of hazards at a non-toxic exposure level. First, the nine different EDCs were tested in cells obtained from two women. Then, those chemicals showing effects (BPA, p,p’-DDE, HCB and PFOS) were studied further in cells from two additional women. Altogether, our results suggest that certain EDCs pose a hazard to endometrial biology. Follow-up studies should focus on detailed characterization of the hazard to allow for risk assessment in the population. It will be essential to study the sensitivity of this endpoint in more detail and develop test systems that are suitable for screening of large numbers of chemicals.

## Materials and methods

### Women, endometrial samples and isolation of stromal cells

Endometrial tissue was collected from the uterus immediately after hysterectomy from four women: endometrial stromal cells (eSCs) coded as 4, 5, 8 and 12, at the gynecological surgical unit at Uppsala University hospital (**Table 2**). All women were in the proliferative phase of the menstrual cycle, had no endometriosis, and were generally healthy (**Table 2**). The tissue was transported to the laboratory on ice in isolation medium: DMEM/F-12 with HEPES without phenol red (Gibco, Thermofisher, USA) containing 1% (v/v) penicillin/streptomycin (Thermofisher, USA). The eSCs 5 and 8 were isolated on the day of surgery. Due to late surgery, eSCs 4 and 12 were kept at 4°C overnight and cells were isolated the next morning.

**Table 2:** Characteristics of tissue donors.

Donor	Age	Fertility	Indication	BMI (kg/m^2^)	Hormone treatment	Smoker	Gravidity	Parity
eSC4	28	unknown	Pelvic pain, especially during menstruation	18	No	No	0	0
eSC5	41	yes	Pelvic pain, especially around and at menstruation	24	No	No	4	3
eSC8	26	yes	Pelvic pain, especially around and at menstruation	26	No	No	2	2
eSC12	41	yes	Menorrhagia and menstrual pain	29	No	No	4	1

The studies were approved by Uppsala Regional ethical review board, Sweden, now the Swedish ethical review authority (reference number 2011/430). Informed written consent was given by all participants in accordance with the declaration of Helsinki. Clinical information was retrieved from patient journals. All samples and data were pseudonymized. Data processing was done in agreement with relevant guidelines (the Swedish data protection law PUL and the General Data Protection Regulation GDPR).

For eSC isolation, the endometrial tissue was placed in a 50 mL tube with isolation medium and isolated as previously described with some adjustments (37–39). Tissue was washed three times in isolation media and cut in smaller pieces using a scalpel. Tissue digestion was done by a mixture of 5 mg/mL collagenase type IA (Sigma Aldrich, Germany) and 0.1 mg/mL DNase I (Roche, Sigma Aldrich, Germany) in isolation media for 1 h at 37°C on a rocking table. After enzymatic tissue digestion, the tube was left to stand for 10 min at room temperature (RT) for sedimentation. The supernatant with eSCs was filtered through a 40 µm cell strainer into a 50 mL collection tube. Sedimentation and filtering were repeated three times. Thereafter, cells were pelleted by centrifugation at 200 × *g* RT.

The cells were then resuspended in 1 mL of DMEM/F12 with phenol red (Gibco, Thermofisher, USA) containing 10% fetal bovine serum (FBS; Thermofisher, USA), 4% Amniomax C100 (Gibco, Thermofisher, USA), 0.2% glutamine (Gibco, Thermofisher, USA) and 0.2% penicillin-streptomycin (growth medium). Erythrocytes were lysed using 90% ACK Lysing Buffer (Gibco, Thermofisher, USA) with 10% growth medium. The eSCs were pelleted by centrifugation (200 x *g* for 6 minutes RT) and cultured in growth medium in a humidified incubator at 37°C with 5% CO_2_ in air.

After reaching ca 80% confluency, cells were detached with trypsin-EDTA (Gibco, Thermofisher, USA) pelleted and resuspended in recovery cell culture freezing medium (Thermofisher, USA) to freeze the cells. MR Frosty (VWR, Avantor, USA) was used with 2-propanol stored at 4°C to achieve a -1°C/min rate of cooling. Cells were slowly frozen down to -80°C overnight and then stored in liquid nitrogen until used.

### Characterization of eSCs

Purity of the eSC suspensions was characterized by immunostaining for stromal and epithelial cell markers using the UltraVision One Detection System HRP Polymer kit (Fisher Scientific TL-060-HLJ) for a chromogenic reaction. The primary antibody anti-vimentin clone V9 (DAKO, Agilent, USA) at 1:250 dilution was used to stain stromal cells, while the primary antibody anti-cytokeratin-8/18 clone Zym5.2 (Thermofisher, USA) at 1:100 dilution was used to stain epithelial cells. Mouse IgG1 (Dako, Agilent, USA) at 1:100 dilution served as a negative control. 100 000 cells/well were plated in an 8-well chamber slide and incubated overnight. The cells were fixed with 4% buffered formaldehyde solution and permeabilized with cold ethanol at -20°C. Hydrogen peroxide block was performed with 0.3% H_2_O_2_ in methanol. Immunostaining was done according to manufacturer’s protocol and the results were recorded with light microscopy.

### Preparation of EDC stocks

HCB, p,p’-DDE, and PCB180 (Sigma-Aldrich, Cat Nos. 45522, 35487, 35495) were dissolved in dimethyl sulfoxide (DMSO, Sigma-Aldrich, Cat No. D8418) to 1-5 mM concentrations by heating in a Thermoblock at 65°C for 15-60 min with intermittent vortexing. Thereafter, the chemicals were sonicated in a water bath for 15-60 min. PCB170 (Sigma-Aldrich, Cat No. 34107), PFOS (Sigma-Aldrich, Cat No. EHERC15987120), PFOA (Sigma-Aldrich, Cat No. 171468) and BPA (Sigma-Aldrich, Cat No. 239658) were dissolved in DMSO to the 1-5 mM concentrations by vortexing. Prof. Christian Lindh (Division of Occupational and Environmental Medicine, Lund University, Sweden) provided 1 M solutions of BPF and MEHP in DMSO. All stock solutions were diluted to 1 mM nominal concentration using DMSO prior to use in cell culture experiments. The stocks were stored in borosilicate glass vials. The persistent chemical stocks (HCB, p,p’-DDE, PCB180, PCB170, PFOS and PFOA) were stored at 4°C and other chemicals (BPA, BPF and MEHP) at -20°C. For mass-spectrometric validation of the concentrations, the DMSO stocks were diluted in starvation medium to achieve 2 μM nominal concentrations. Concentrations of HCB, p,p’-DDE, PCB170 and PCB180 in the DMSO stocks were validated using gas chromatography – tandem mass spectrometry (GC-MS/MS) by following a previously published method (40). MEHP, BPF, BPA, PFOS and PFOA concentrations were directly analysed from the DMSO stocks without any pretreatment by previously published liquid chromatography tandem mass spectrometry (LC-MS/MS) methods (40–42). Data of these validation analyses are shown in Supplement (**Table S1**).

### Treatment and lysis of eSCs

Prior to the *in vitro* decidualization studies, isolated eSCs were cultured for 2 passages in assay medium: Dulbecco’s Modified Eagle’s medium (DMEM)/Ham’s F12 medium without phenol red (Sigma-Aldrich, Steinheim, Germany) supplemented with 10% charcoal-purified fetal bovine serum (ccFBS), 2 mM L-glutamine (both from Sigma-Aldrich; Steinheim, Germany) and a mixture of penicillin (100 U/mL), streptomycin (100 μg/mL), and amphotericin B (0.25 μg/mL; from Capricorn, Ebsdorfergrund, Germany). After reaching confluency, the cells were seeded onto 6-well plates with the density of 100 000 – 170 000 cells per well; after 1-3 days, the assay medium was replaced with starvation medium (containing the same components as the assay medium, but 2% ccFBS), and after additional 24 h, treatment was started. Decidualization mixture (DC-mix) hormones and chemicals were added to the starvation medium to obtain the following final concentrations: 10 nM β-estradiol (E2); 100 nM progesterone (P4); 200 μM 8-bromoadenosine 3′,5′-cyclic monophosphate sodium salt (8-Br-cAMP); and 100 μM 3-isobutyl-1-methylxanthine (IBMX). All DC-mix components were from Sigma-Aldrich (Steinheim, Germany); the corresponding solutions were prepared in cell culture grade DMSO (AppliChem; Darmstadt, Germany) as 1000-fold concentrated stocks (1 000x), and stored at -20°C. The EDCs were added at 1 μM final concentration. By volume, the final concentration of the vehicle (DMSO) in the treatment mixtures was as follows: 0.1% in case of E2 control, 0.4% in case of DC-mixture, and 0.5% in case of DC-mixture + any EDC. The *in vitro* decidualization protocol lasted for a total of 9 days; the treatment mixtures were replaced every 72 h (full medium change).

Spent culture media were collected and stored at -80°C. At the end of the 9-day treatment, the cells were rinsed with phosphate-buffered saline (PBS; from Sigma-Aldrich, Steinheim, Germany) and lysed as previously reported (34). The lysis buffer components were from the following sources: HEPES and NaCl – Calbiochem (Darmstadt, Germany); EDTA – Scharlau (Barcelona, Spain); Triton X-100 – Ferak (Berlin, Germany); phenylmethylsulfonyl fluoride (PMSF) – AppliChem (Darmstadt, Germany); cOmplete™ protease inhibitor cocktail – Roche (Basel, Switzerland). After 1 h lysis on ice, the membranes were pelleted by centrifugation, and the supernatants were collected.

### Assessment of viability

Cell viability and proliferation were estimated by quantifying total protein concentration in lysates and by measuring the activity of the viability marker CK2 at the end of the 9-day decidualization assay. The total protein concentration in cell lysates was measured using Bradford assay utilizing Pierce™ Coomassie Plus (Bradford) Assay Reagent (Thermo Fischer Scientific; Rockford, IL, USA) according to the manufacturer’s instructions. The bovine serum albumin (BSA) dilutions (2 - 128 µg/mL) used for calibration and PBS (supplemented with Ca^2+^, Mg^2+^) used as a negative control for the Bradford assay were from Sigma-Aldrich (Steinheim, Germany). The absorbance was measured with PHERAstar multi-mode reader (BMG Labtech; Ortenberg, Germany) with the following parameters: filter block 608A (590 nm), 20 flashes per well, focal height 10.5 mm, optical pathway correction for volume of 150 μL.

The total protein concentration in each sample was then normalized to the same level between the samples lysed on the same day using the kinase assay buffer (50 mM HEPES pH 7.5, 150 mM NaCl, 0.005% Triton X-100, 0.5 mg/mL BSA, 5 mM dithiothreitol). For measuring activity of CK2, the previously described protocol was used (34). Briefly, ARC-1530 probe (final total concentration of 2 nM) was added to the lysate dilutions (total protein concentration of 200-400 μg/mL). ARC-1530 was synthesized as reported previously (43). Following 60 min incubation, the photoluminescence of the probe was measured with PHERAstar multi-mode reader (BMG Labtech; Ortenberg, Germany) using the following parameters: time-delayed photoluminescence, excitation 337 (300–360) nm, emission 590 (50) nm, 200 flashes per well, integration start 60 μs, integration time 400 μs. Next, the selective competitive CX-4945 (from Synkinase, Shanghai, China) was added (final total concentration of 2-4 μM); after 30-60 min incubation to achieve equilibrium, photoluminescence was measured again.

### Measurement of decidualization markers

Decidualization was measured by quantification of PRL in spent culture media samples (from the last 72 h of the *in vitro* treatment) and assessment of kinase activities in cell lysates following the 9-day *in vitro* decidualization as described (34). In media samples, the total protein concentration was measured as described above. Then, PRL concentration was measured using human PRL ELISA kit (Cayman Chemical; Ann Arbor, MI, USA); the absorbance readings at 450 nm were measured using Cytation 5 multi-mode reader (Biotek; Winooski, VT, USA). Finally, the PRL concentration calculated from the calibration curve was normalized to the total protein concentration in the same media aliquot.

For spent culture media samples collected in experiments with p,p’-DDE and HCB-containing mixtures, levels of the IGFBP-1 were also assessed for the non-decidualized control cells, decidualized cells (9-day treatment with DC-mix), and cells treated for 9 days with DC-mix supplemented with 1 μM p,p’-DDE or HCB. IGFBP-1 concentration was measured using human IGFBP1 ELISA kit (Invitrogen; Waltham, MA, USA); the absorbance readings at 450 nm were measured using Cytation 5 multi-mode reader (Biotek; Winooski, VT, USA). Again, the IGFBP-1 concentration calculated from the calibration curve was normalized to the total protein concentration in the same media aliquot.

The activities of protein kinases PKAc, ROCK, and Akt/PKB were assessed using a competitive photoluminescent displacement assay based on kinase-responsive probes as described (34). After the lysis of cells and normalization of the total protein concentration (measured by Bradford as described above) to the same level between the samples lysed on the same day, ARC-1139 probe (final total concentration of 2 nM) was added to the lysate dilutions (total protein concentration of 200-400 μg/mL); ARC-1139 was synthesized as reported previously (44). Following 60 min incubation, the photoluminescence of the probe was measured with PHERAstar multi-mode reader (BMG Labtech; Ortenberg, Germany) using the following parameters: time-delayed photoluminescence, excitation 337 (300–360) nm, emission 675 (50) nm, 100 flashes per well, integration start 60 μs, and integration time 400 μs. Next, the selective competitive kinase inhibitors H89 (PKAc), Y-27632 (ROCK) or GSK690693 (Akt/PKB) were added (final total concentrations of 2-4 μM, 2-4 μM and 0.2-0.4 μM, respectively); after 30-60 min incubation to achieve equilibrium, photoluminescence was measured again. Protein kinase inhibitors were obtained from the following sources: H89 – Biaffin (Kassel, Germany); Y-27632 – Cayman Chemical (Ann Arbor, MI, USA); GSK690693 – Tocris (Bristol, UK).

### Effects of EDCs and control compounds on kinases in cell-free conditions

In order to address whether the EDCs might directly bind the protein kinases thereby affecting their activities, cell-free binding assays were carried out with recombinant PKAcα (human full-length protein; Biaffin), ROCK2 (human His_6_-tagged protein, amino acids 11-552; Millipore), Akt3/PKBγ (human His_6_-tagged S472D mutant protein, amino acids 117-C-terminus; Millipore), and CK2α (human protein, amino acids 1-335; a kind gift from Prof. Karsten Niefind, University of Cologne) according to the previously established protocols (43–45). Briefly, solutions of EDCs or control inhibitors in kinase assay buffer were prepared and a complex consisting of a kinase and a fluorescent or photoluminescent probe was added; the signals were measured following 30-60 min incubation (**Table S2**). In case of PKAcα, two different probes were used to ensure the sufficient dynamic range of assay. The final total concentrations of EDC in the reaction mixtures were 1 μM.

The selectivity of the chosen inhibitors within the set of tested kinases was confirmed using a similar format, yet the final concentrations of the probes (2 nM) were chosen to mimic the conditions used in the lysate assays. The final total concentrations of active kinases in the assay mixture were equal to 3 nM. For assessment of the dose-response profile, the dilution series of displacing compounds (3-fold dilutions starting from 20 μM final concentration) were tested (**Figure S1**). Two independent experiments were carried out.

### Effect of EDCs on ELISA and Bradford assay components

To assess whether EDCs alone have impact on the measurement of PRL, elevated concentrations of EDCs (5 µM in the final assay mixture) were individually spiked into the 100 ng/mL PRL standard included in the assay kit, and the ELISA was further carried out according to the manufacturer’s instructions. As a control, 100 ng/mL PRL standard spiked with 0.5% DMSO was used. The experiment was performed once in duplicates.

To assess whether EDCs alone have impact on the measurement of the total protein content, elevated concentrations of EDCs (5 µM in the final assay mixture) were individually spiked into the PBS buffer, and two-fold dilution series of a standard calibration protein BSA into the spiked buffers were made. As a control, analogous dilution of BSA was carried out in the non-spiked PBS. The Bradford assay was subsequently carried out according to the manufacturer’s instructions. The experiment was performed once in duplicates.

### Statistical analysis

For general data analysis, GraphPad Prism 9.2.0 (San Diego, CA, USA) and Excel 2016 (Microsoft Office 365; Redmond, WA, USA) were used.

In case of PRL ELISA, the normalized PRL content (in ng/mg total protein) was pooled for samples treated in the same conditions in each independent experiment (please note that several vials of cells were available for samples from each patient, so more than one independent experiment could be made using eSCs from one patient: N = 5 for eSC4; N = 5 for eSC5; N = 7 for eSC8; N = 4 for eSC12; all measurements performed in duplicates). For assessment of effect of EDCs on decidualization, additional normalization was carried out by setting the normalized PRL content in the DC-mix to 100% in each independent experiment, prior to pooling of data. The statistical significance of differences between the DC-mix *versus* other groups was established for pooled data by one-way ANOVA with Dunnett’s test for multiple comparisons. For assessment of EDC effect on the ELISA kit components, the apparent concentrations of spiked standards were calculated using the calibration curve. The statistical significance of difference between the 0.5% DMSO control *versus* other groups was established by one-way ANOVA with Dunnett’s test for multiple comparisons.

In case of IGFBP-1 ELISA, the experiment was carried out using samples from a total of four independent experiments: a single experiment with eSC4, a single experiment with eSC5, and two experiments with eSC8. Normalization of data was carried out as in case of PRL ELISA. The statistical significance of differences between the DC-mix *versus* other groups was established for pooled data by one-way ANOVA with Dunnett’s test for multiple comparisons.

In case of protein kinase activity assay in lysates, for each target kinase the difference of probe signal prior to and after addition of competing inhibitor was calculated. For assessment of effect of EDCs on decidualization, the values calculated for each target kinase in different lysates within a single independent experiment were normalized to the value calculated for lysate from eSCs treated with the DC-mix (activity set to 100%). The results of all measurements were then pooled (N = 5 for eSC4; N = 5 for eSC5; N = 6 for eSC8; N = 4 for eSC12; all measurements performed in duplicates). The statistical significance of differences between the DC-mix *versus* other groups was established for pooled data by one-way ANOVA with Dunnett’s test for multiple comparisons.

In case of recombinant protein kinase activity assay, the probe signal measured for the inhibitor-containing mixtures was normalized to the signal of kinase-probe complex (activity = 100%) and free probe (activity = 0%). The results of all measurements were then pooled (N = 2 for each kinase; all measurements performed in duplicates). The statistical significance of difference between the kinase-probe complex *versus* inhibitor-containing mixtures was established for pooled data by one-way ANOVA with Dunnett’s test for multiple comparisons. Data is presented as mean with standard error of mean (SEM). P-values <0.05 were considered significant and are marked in the figures as follows: *P<0.05, **P<0.01, ***P<0.001.

## Results

### Study design

**Figure 1:**
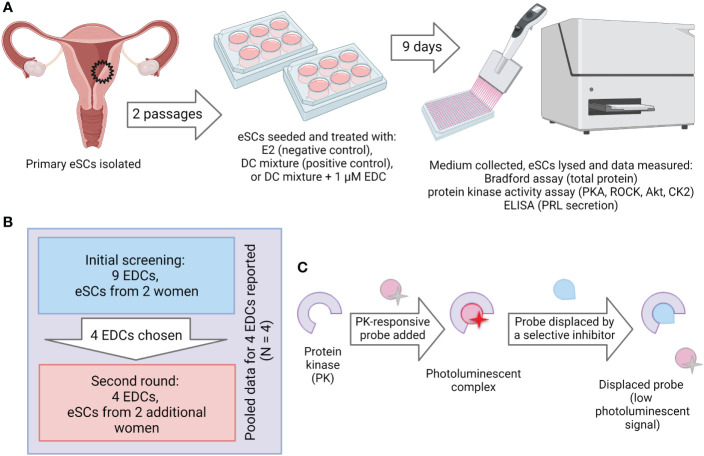
Experimental setup. **(A)** Workflow of the decidualization disruption assay. Human endometrial tissue samples were digested to single-cell suspension and endometrial stromal cells (eSCs) were isolated. The eSCs were cultured for two passages before they were cryopreserved for later use in experiments. To induce decidualization, the cells were exposed to decidualization mixture (DC-mix) composed of estradiol (E2), progesterone (P4), 8-Br-cAMP and IBMX for nine days. To test the effect of EDCs on decidualization, the cells were co-exposed to DC-mix and 1 μM of EDCs. Decidualization was assessed by measuring prolactin (PRL) secretion in culture media and activation of protein kinases PKA, ROCK and Akt in lysates of treated cells. Effects on cell proliferation and viability were estimated by the total protein concentration and activity of the viability marker CK2 in lysates of treated cells. **(B)** Initial screening was performed with nine EDCs on eSCs from two different women. From these nine chemicals, four were chosen for the exposure studies with ESCs from two additional women. **(C)** The principle of the protein kinase activity assay in lysates. Binding of a generic kinase probe to the mixture of target kinases present in lysate samples produces long-lifetime photoluminescent signal. Activity of each protein kinase of interest is subsequently established by displacing the probe with a competing selective inhibitor (H89 in case of PKA, Y-27632 in case of ROCK, GSK-690693 in case of Akt, and CX-4945 in case of CK2).

Primary uterine eSCs were used to study the effects of EDCs on decidualization *in vitro* using kinase activities and PRL secretion as endpoints (**Figure 1**). We selected nine EDCs based on their ubiquitous presence in biomonitoring samples from reproductive age women (46), as well as their significant associations to lower fertility of possible endometrial origin (longer time-to-pregnancy, miscarriage, pre-eclampsia) (17–19, 35). Some of the chosen EDCs were non-persistent (BPA, BPF, MEHP) and others persistent with lipophilic (p,p’-DDE, HCB, PCB170, PCB180) or amphiphilic (PFOS, PFOA) properties. EDC stocks were prepared in DMSO, and the concentrations were validated by mass spectrometry (**Table S1**). All stocks were confirmed to have concentrations close to expected (80 – 130%) except for the two PCBs that had markedly lower concentrations than anticipated: PCB170 was 20% and PCB180 was 45% of the expected concentration (**Table S1**). PCBs are notoriously difficult to dissolve, even by heating, vortexing and sonication. Here, nominal concentrations will be used when results are presented.

Endometrial cells were isolated from hysterectomized uteri in proliferative stage from non-smoking women aged 26 to 41 years not using hormonal contraception. They underwent hysterectomies due to pelvic pain and menorrhagia (**Table 2**). The purity of the endometrial stromal cells was studied by immunostaining and found to be satisfactory (**Figure S2**). The decidualization protocol was optimized in our previous study and consisted of a nine-day exposure of the cells to the DC-mix composed of 8-Br-cAMP, P4, E2 and IBMX (34). 8-Br-cAMP has been used in multiple *in vitro* decidualization protocols because it activates the cAMP/PKA pathway that is physiologically presumably initiated by prostaglandin E2 (PGE2) (47) or relaxin receptor-mediated signaling (48). However, we have shown that 8-Br-cAMP alone is not sufficient but requires the presence of P4 to trigger the whole spectrum of changes occurring during the transition from the proliferative to the secretory endometrium (34). Additionally, we added IBMX to prevent the PDE-catalyzed hydrolysis of 8-Br-cAMP, and E2 to reflect the constant presence of the hormone in women. The duration of the decidualization protocol was optimized in our previous studies where we demonstrated that shorter treatment (4 days) only resulted in a partial development of the decidualization-characteristic fingerprint of markers, whereas the prolonged 9-day protocol enabled us to achieve higher measurement windows for all markers (34).

### EDCs are not cytotoxic

To verify that the selected concentrations are not cytotoxic to the cells, cell proliferation and viability were estimated by measuring protein concentration and activity of the cell proliferation kinase CK2 in cell lysates after the nine-day culture. CK2 is a well-characterized pro-survival kinase that sustains viability of cells by phosphorylating the major chaperone machinery represented by the Cdc37-Hsp90 complex (49); the reduced activity of CK2 thus mirrors reduction in number of viable cells in the sample. Compared to DC-mix, none of the EDCs significantly reduced protein content or CK2 activity, suggesting that the exposures were not cytotoxic (**Figure S3**). Only MEHP increased protein content, which may indicate increased proliferation.

### EDCs disrupt markers of decidualization *in vitro*

**Figure 2:**
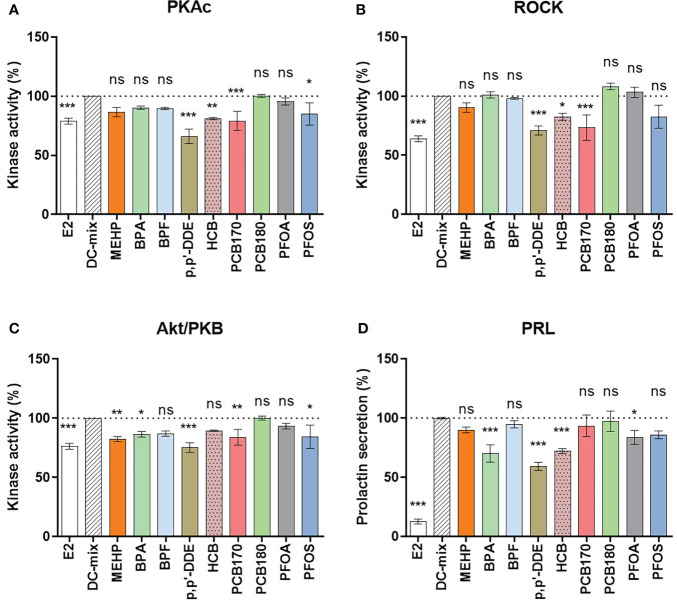
Screening of nine EDCs for effects on decidualization. The eSCs of two women (N=2) were exposed to the decidualization-inducing mixture (DC-mix) in the absence or presence of 9 different EDCs (1 µM) for 9 days, and 10 nM E2 was used as a negative control. Altogether 10 independent assays were carried out (n=10). Protein kinase activity was assessed in lysates (normalized to protein content) using a competitive photoluminescent probe displacement assay, and calculated as percentage of DC-mix (100%) for **(A)** PKAc, **(B)** ROCK and **(C)** Akt/PKB. **(D)** Secreted PRL was measured in spent media representing the last 72 h of the in vitro treatment using a human PRL ELISA kit. Values are expressed as mean percentage of DC-mix alone ± SEM (N = 2, n = 10). Significance is calculated by one-way ANOVA with Dunnett test for multiple comparisons (95% CI) relative to DC-mix and indicated as *P<0.05, **P<0.01 and ***P<0.001 and ns, not significant. The dotted line corresponds to 100%.

In the first step, the nine EDCs were screened for effects using eSCs from two women. The cells were treated with DC-mix for 9 days with or without EDCs present at 1 μM concentrations. Media were changed every three days, and effects on kinases and PRL were measured in lysates or in media aliquots collected on the last day of culture. Expectedly, the DC-mix significantly increased PRL secretion and activity of all kinases compared to E2 treatment alone (**Figure 2**). In all experiments, DC-mix was thus used as positive control and set to 100%, and 10 nM E2 alone was used as negative control. Our previous experiments have shown that E2 and DMSO are comparable negative controls in this system (34) and we chose to use E2 here to reflect the constant presence of this hormone in the female reproductive system during fertile years.

When we compared the signal from the positive control (DC-mix) to the experimental groups (DC-mix+EDC) we found that some EDCs significantly reduced markers of decidualization. BPA, p,p’-DDE, HCB and PFOA significantly reduced the secretion of the classical decidualization marker PRL in culture medium to 70 ± 8%, 59 ± 4%, 72 ± 2% and 84 ± 6% (**Figure 2**). These effects were associated with significantly reduced kinase activities although not consistently: BPA caused reduced Akt activity (86 ± 3%); p,p’-DDE caused reduced PKAc (66 ± 6%), ROCK (71 ± 4%) and Akt (75 ± 4%) activities; HCB caused reduced PKA (81 ± 1%) and ROCK (83 ± 3%) activities; and PFOA did not significantly affect activity of any kinases. Interestingly, PFOS and PCB170 also caused reduced kinase activities although these EDCs did not have statistically significant effects on PRL secretion. However, it can be observed that PFOS exposure also reduced PRL secretion, but the effect did not reach significance at p<0.05 (p=0.052). Finally, BPF and PCB180 did not have any significant effects on the kinase activities or PRL secretion. Collectively, EDCs representing different physicochemical properties significantly reduced hormone-stimulated decidualization process *in vitro*. Reduced PRL secretion, a hallmark of decidualization residing downstream of the cAMP/PKA signaling (50), was accompanied with reduced kinase activities although certain variation was observed, suggesting that multiple upstream pathways can lead to disrupted PRL secretion.

To confirm the observed trends, we additionally assessed whether secretion of yet another decidualization biomarker, IGFBP-1, was affected by the presence of p,p’-DDE or HCB in the treatment mixture (**Figure S4**). These analyses confirmed a statistically significant reduction of IGFBP-1 secretion in the presence of EDCs. Normalized secretion of 81 ± 5% (p<0.01 relative to DC-mix) and 84 ± 8% (p<0.05 relative to DC-mix) was measured for the cells treated with mixtures containing p,p’-DDE and HCB, respectively.

### EDCs do not directly inhibit kinases

**Figure 3:**
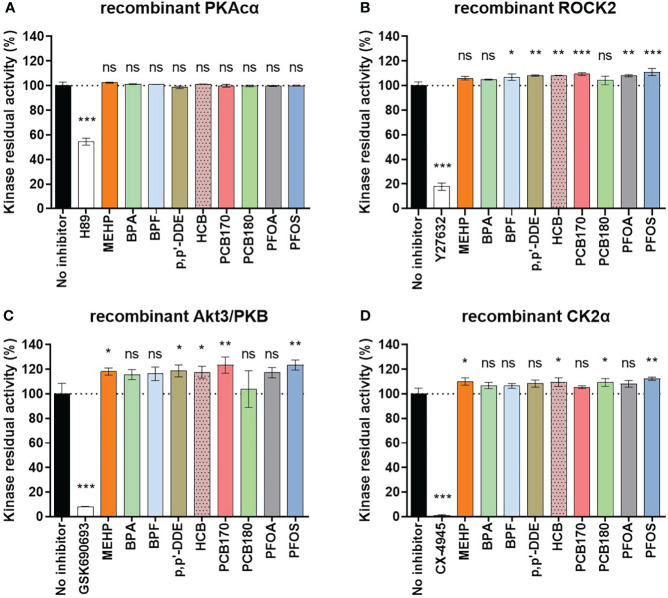
Effect of EDCs (1 μM) and selective control inhibitors (1 μM) on activities of recombinant protein kinases to assess whether EDCs have a direct effect on the protein kinases. Recombinant protein kinases **(A)** full-length PKAcα; **(B)** His_6_-ROCK2 (amino acids 11-552); **(C)** His_6_-Akt3 (S472D mutant, amino acids 117-end); **(D)** CK2α (amino acids 1-335) were used. The graph shows the averaged data of the three measurements as mean ± SEM (n = 2 for each kinase, performed in duplicates). Significance is calculated by one-way ANOVA with Dunnett test for multiple comparisons (95% CI) relative to no inhibitor and indicated as *P<0.05; **P<0.01 ***P<0.001 and ns, not significant. The dotted line corresponds to 100%.

We also wanted to study whether the EDCs at 1 μM concentration might directly interfere with the activity of the kinases. For this, cell-free kinase binding assay was performed using recombinant protein kinases. As controls, selective inhibitors of the corresponding protein kinases were used (H-89 in case of PKAcα, Y27632 in case of ROCK2, GSK690693 in case of Akt3, and CX-4945 in case of CK2α; the selectivity of the corresponding inhibitors was validated by the dose-response studies of each compound with all four protein kinases of interest, see **Figure S1**). Expectedly, the selective inhibitors for all four kinases strongly reduced kinase residual activity (**Figure 3**). None of the EDCs affected activity of recombinant PKAcα (**Figure 3A**). Interestingly, activities of the other kinases were slightly upregulated by the EDCs. Compared to the control set to 100%, the activity of recombinant ROCK2 was increased by several EDCs (BPF 107 ± 2%; p,p’-DDE 108 ± 1%; HCB 108 ± 0%; PCB170 109 ± 1%; PFOA 108 ± 1%; PFOS 111 ± 2%), activity of recombinant Akt3 was increased by MEHP (118 ± 2%), p,p’-DDE (119 ± 4%), HCB (118 ± 4%), PCB170 (124 ± 5%) and PFOS (124 ± 3%), and that of recombinant CK2α increased by MEHP (110 ± 2%), HCB (110 ± 3%), PCB180 (109 ± 3%) and PFOS (112 ± 1%). This could be explained by reduction of non-specific binding of the kinase or the photoluminescent probe in the presence of EDCs containing hydrophobic moieties. BPA did not affect any of the recombinant protein kinases. Overall, all observed differences showed increased activity, not decreased, which suggests that the effects of EDC on reduced kinase activities in eSC lysates do not originate from direct inhibition of the kinases but rather from overall impact on hormone-triggered signal transduction leading to decidualization. The decidualization marker kinases explored here (PKAc, ROCK and Akt) all require upstream activation (by cAMP, Rho and PI3K/PDK1, respectively) which adds further levels of complexity into the model of decidualization disruption by EDCs. The exact molecular targets of EDCs need to be explored in future studies, for instance by utilizing large-scale profiling techniques such as proteomics, transcriptomics, or metabolomics. Knowledge of precise mechanisms could help to develop novel assays for decidualization that are not dependent on primary patient materials.

### Validation of BPA, p,p’-DDE, HCB and PFOS effects

**Figure 4:**
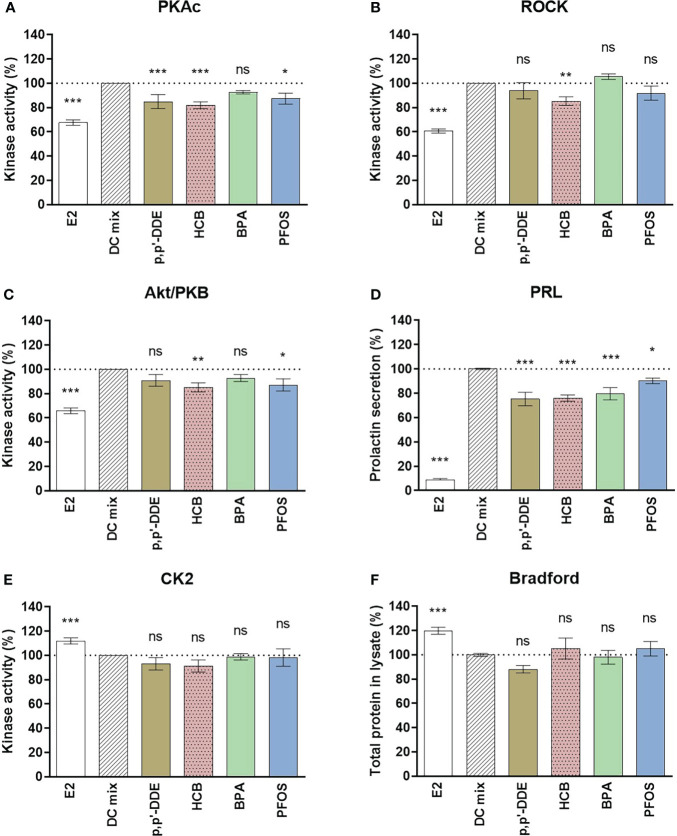
Effect of DDE, HCB, BPA and PFOS on decidualization and viability markers. The eSCs of two additional women were exposed to the decidualization-inducing mixture (DC-mix) in the absence or presence of 1 µM DDE, HCB, BPA, PFOS for 9 days. The effects of EDCs were compared to the positive control DC-mix, and 10 nM E2 was used as negative control. Total protein concentration for different treatments was normalized after lysis of the eSCs. Protein kinase activity for **(A)** PKAc, **(B)** ROCK2 and **(C)** Akt/PKB was assessed in lysates using a competitive photoluminescent probe displacement assay and calculated as percentage of DC-mix (100%). **(D)** Secreted PRL was measured in media aliquots (collected following the last 72 h of the in vitro treatment) using a human PRL ELISA kit. The impact of the exposures on viability of the endometrial stromal cells in culture during decidualization assays was assessed by the activity of the viability marker kinase CK2 in lysates **(E)** as well as evaluating the total protein concentrations in lysates at the end of the assay **(F)**. Values are expressed as mean percentage of DC-mix alone ± SEM based on cells derived from 4 women (N=4) tested in 4 to 6 independent experiments (n=4-6). Significance is calculated by one-way ANOVA with Dunnett test for multiple comparisons (95% CI) relative to DC-mix and indicated as *P<0.05, **P<0.01 and ***P<0.001 and ns, not significant. The dotted line corresponds to 100%.

Additional replicate experiments were carried out with BPA, p,p’-DDE, HCB and PFOS using cells from two more women to validate the results. The pooled results of the experiments with samples from four women suggest that these chemicals significantly disrupt hormone-induced decidualization *in vitro* (**Figure 4**). Even though there was variation in responses between the samples (**Figure S5**), statistical analyses showed that all four EDCs significantly reduced secretion of PRL into culture medium (p,p’-DDE 75 ± 6%, HCB 76 ± 3%, BPA 80 ± 5%, PFOS 90 ± 3% compared to DC-mix set to 100%). Considering markers of cell viability and proliferation, there was a significant difference between the negative control E2 and positive control DC-mix for both CK2 activity and the total protein content (**Figure 4**), which was consistent with our previously reported observations regarding the reduced proliferation rate of eSCs upon treatment with DC-mix (34). Furthermore, consistent with the screening of the nine different EDCs (**Figure S3**), viability of the cells was not significantly affected by EDC exposure (**Figure 4**). The variability between samples from different women in response to hormone-stimulated decidualization *in vitro* was also noted in our previous studies utilizing primary cells (34, 51) and this, at least in part, reflects varying sensitivity of individuals to hormones (components of the DC-mix) and EDCs. The reduced PRL secretion in cell culture media was accompanied by varying degrees of reduction in kinase activities. BPA had the least effects on kinases (none significantly affected in the pooled data), p,p’-DDE caused reduced PKA activity (85 ± 6%), PFOS caused reduced PKA (87 ± 5%) and Akt (87 ± 5%) activities, and HCB affected all markers (82 ± 3% PKA; 85 ± 4% Akt; 85 ± 4% ROCK).

**Figure 5:**
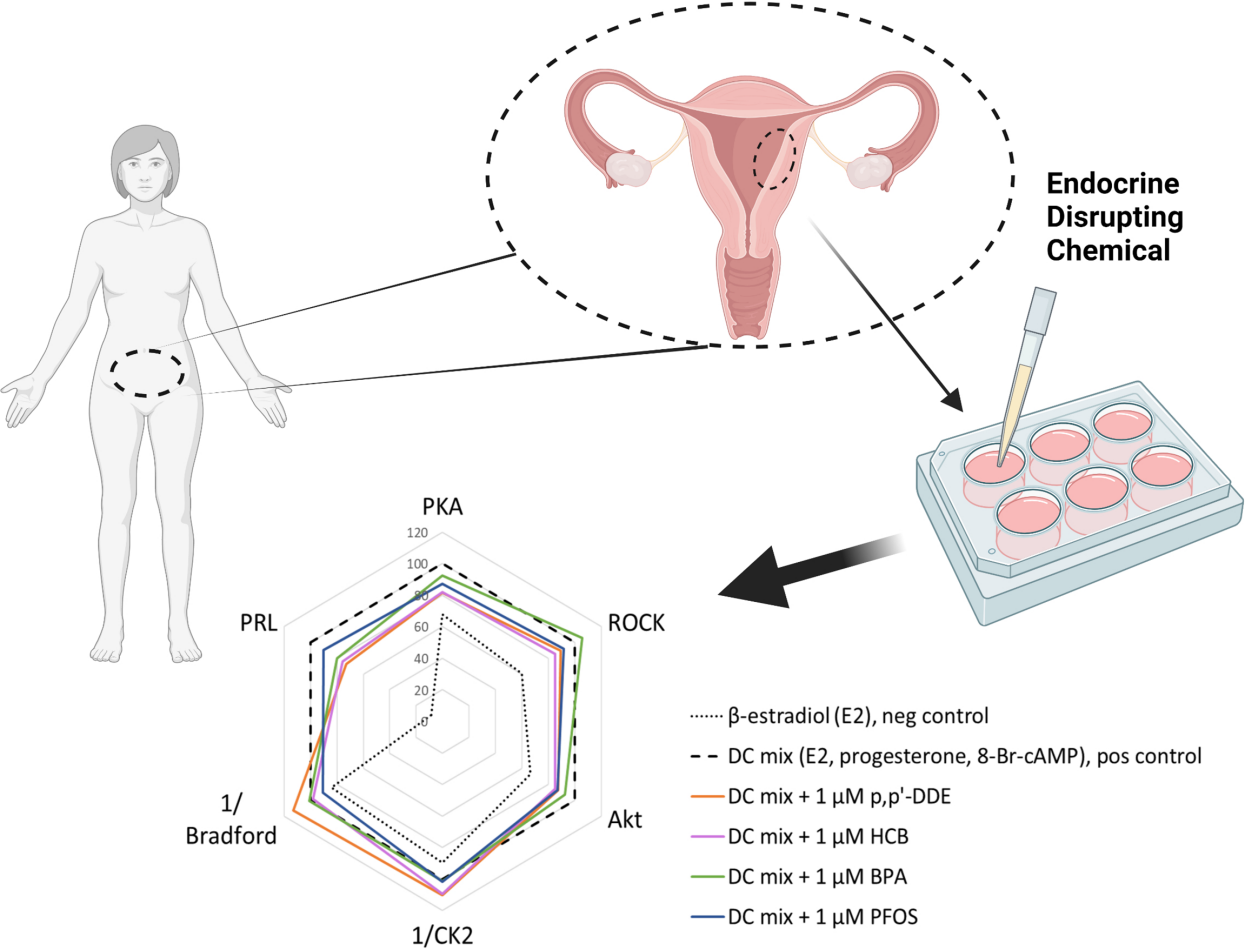
Summary of the 1 μM EDC impact on decidualization markers following in vitro treatment of ESCs. The radar plot depicts a characteristic ‘fingerprint’ of various decidualization- and viability-related markers measured in this study and the alterations caused by the presence of EDCs (as seen by shift of pattern related to that of the DC-mix). The y-axis shows normalized activities (%) of PKA, ROCK and Akt, normalized secretion (%) of prolactin (PRL), normalized reciprocal of CK2 activity (%) and normalized reciprocal of total protein content (indicated as Bradford) (%). In each case, 100% corresponds to the value measured for the decidualization mixture; the reciprocal values for CK2 activity and total protein content reflect the fact that these markers are reduced during decidualization. The graph summarizes data from all independent experiments performed within the study (N = 4 and n = 21 for each EDC); for better clarity, error bars are not shown.

To exclude that the measurements would be affected by EDCs interfering with PRL or protein concentrations measurements in general, additional control experiments were carried out by spiking PRL assay standard and BSA (Bradford assay standard) with 5 μM EDCs. A slight reduction of approximately 10% in PRL by 5 μM DDE and HCB was observed (**Figure S6**). This can be considered as a minor effect compared to the 25% decrease in PRL secretion measured in the cell assays at the 5-fold lower concentrations of the EDCs (**Figure 4**). There were no effects on the Bradford assay. In conclusion, the impact of the tested EDCs on markers of decidualization of human eSCs *in vitro* is likely not due to technical reasons, but reflects disruption of hormone-stimulated cellular signal transduction underlying decidualization process. The shift in the decidualization marker ‘fingerprint’ pattern of the DC-mix caused by addition of 1 μM p,p’-DDE, HCB, BPA, or PFOS is summarized in **Figure 5**.

## Discussion

There is a lack of relevant model systems for screening and characterization of chemicals that disrupt fertility in women (2, 8, 52). It is clear that elevated EDC serum levels correlate with worse female fertility in cohort studies (17–20, 35), but since correlation does not mean causation, experimental studies are needed to provide the proof for causality, to identify most sensitive target organs, and also to understand mechanisms behind their effect. Human primary eSCs can be obtained from hysterectomies, isolated, and studied in culture. Such cells are hormone-sensitive and recapitulate many aspects of endometrial biology, and have been used for example to study contraceptive effects on embryo attachment *in vitro* (37, 53–55), or even applied to EDC research (**Table 1**). To the best of our knowledge, our study is the first one testing a large set of EDCs, at relatively low levels, in primary cells. Although clinical primary samples are prone to substantial inter-individual variation, like observed in our studies (**Figure S5**), we found significant disruption of decidualization by EDCs using cells from four women. The EDC concentrations tested were higher than typically found in women or reproductive age, but so was the concentrations of steroid hormones in the DC-mix supraphysiological too. As such, the results from our model show that the process of decidualization is sensitive to EDCs of various physico-chemical properties. Future studies should test whether immortalized cell lines could be used to detect same effects as this would allow wider exploitation of the decidualization model in a broader range of chemicals. On the other hand, based on our previous experience with immortalized cell lines *vs* primary cells, the profile of markers related to the *in vitro* decidualization is substantially different, posing questions regarding the adequacy of applicability of the immortalized cell lines for such studies.

Among the markers assessed here, PKAc is a canonical player in decidualization, being activated by the increasing intracellular levels of cAMP and triggering changes in gene expression *via* CREB and other transcription factors (9, 50). The reports on the status of Akt signaling in decidualization are somewhat conflicting, as several groups have previously reported reduction in phosphorylation levels and thus activity of Akt during *in vitro* decidualization (56); our previous studies have, however, shown increase in Akt activity (34). We presume that Akt might be involved in metabolic reprogramming characteristic for the transition from proliferative to secretory endometrium; furthermore, other studies (57, 58) have also highlighted the importance of anti-apoptotic effects of Akt for the successful decidualization, embryo implantation and pregnancy onset. ROCK was identified in our previous study (34) as a novel downstream target of progesterone; in the context of decidualization, ROCK is responsible for the stabilization of cytoskeleton and changes in the migratory properties of eSCs associated with the mesenchymal-epithelial transition (59).

The exact decidualization-relevant targets affected by the EDCs are beyond the scope of this study. The cell-free kinase activity measurements showed that there was no direct inhibitory effect of EDCs on the protein kinases tested. Presumably, modulation of the kinase activities by EDCs can occur on the level of upstream activation – *e.g*., by binding of EDCs to the membrane G-protein couple estrogen receptor that mediates cAMP and Ca^2+^ signalling (60, 61). Moreover, the reduction in decidualization-related kinase markers but not PRL secretion observed in this study (**Figure 2**) for PFOS and PCB170 suggests that PRL expression can be regulated not only *via* PKAc, ROCK or Akt pathways, or that the kinase activity must be reduced below a certain threshold value before reduction of downstream gene expression is triggered. The complexity of the EDC-mediated effects on the level of gene expression is further augmented by the documented interactions of EDCs with the steroid receptors.

eSCs are central to embryo attachment and implantation, and disruption of their function could lead to implantation and/or placentation failure contributing to pregnancy complications and loss of pregnancy (62, 63). Our aim was to study whether decidualization can be disrupted by chemicals commonly found in reproductive age women. The concentration of EDCs chosen (1 μM) is relatively high compared to the population exposure levels (46, 64), yet lower than in several previous studies (**Table 1**). If safety factors are to be considered, effects of EDCs at 1 μM concentration would convert to a 1000-fold lower safe exposure levels (1 nM), which are relevant from the typical human exposure point of view. In reality, solubility issues led to a situation where the levels of PCBs in the experiment were lower than anticipated (**Table S1**); however, they were likely higher than the typical pM levels measured in serum of women. At these conditions, half of the studied EDCs disrupted at least one marker of decidualization *in vitro* (**Figure 5**), which is perhaps not surprising, as we chose them for their known associations to infertility, pregnancy loss and longer time-to-pregnancy in cohort studies (18–20, 35, 36).

A limitation of our study is that we used eSCs from four different women only. However, the most significant trends could be reliably pinpointed already at this statistical level. Regarding the decidualization protocol used here, two limitations can be emphasized. First, the measurements were only performed after 9 days of decidualization, yet we have confirmed before that for the set of monitored markers, such duration of the experiment is optimal (34). Second, we used E2 instead of DMSO as a negative control, yet it was previously shown that either DMSO or E2 are suitable treatments representing the non-decidualized cells in case of the measured set of markers (34); furthermore, we considered application of E2 important in this study, as the EDCs would have to compete with physiological levels of E2 also within the female organism. Yet another limitation is represented by the use of protein kinase inhibitors for dissection of individual kinases (PKA, ROCK, Akt, and CK2), as the selectivity of compounds is not absolute. Still, at least from the aspect of the cross-selectivity within the set of the chosen markers (**Figure S1**), the selectivity of the displacing compounds is sufficient to draw the outlined conclusions.

Altogether, the results suggest that EDCs may pose a hazard to decidualization, and endometrial cells should be considered as a target of endocrine disruption. Notably, endometrium normally renews every month (65), so accumulation of more persistent EDCs in this tissue is unlikely; thus the exposure *via* the blood circulation seems more relevant. We focused on the effects of single EDC exposures. However, women are never exposed to one chemical only, but to complex mixtures of contaminants where combination effects have been shown to occur leading to adverse health consequences (66). Together with the many cohort studies (18–20, 35, 36), our *in vitro* screen suggests that EDC exposure may have causal link to miscarriage and infertility in women. Follow-up studies need to address concentration-response relationship to enable evaluation of risks. The role of EDCs in endometrial biology, implantation failure and miscarriage in women could be studied in more detail with the help of women attending infertility clinics, for example. Finally, by determining how EDCs affect fertility in endometrial samples, adjusted treatments for these women could be implemented leading to more successful pregnancies and live births in the future.

## Data availability statement

The original contributions presented in the study are included in the article/**Supplementary Material**. Further inquiries can be directed to the corresponding author.

## Ethics statement

The studies involving human participants were reviewed and approved by Uppsala Regional Ethical review board. The patients/participants provided their written informed consent to participate in this study.

## Author contributions

DL: protein kinase assay, PRL assay, writing and checking the manuscript. NV: stromal cell isolation, literature analysis, writing and checking the manuscript. KS: cell culturing, checking the manuscript. ED: stromal cell isolation and validation, checking the manuscript. RB: Preparation of chemical stocks, checking the manuscript.. JH: Preparation of chemical stocks, checking the manuscript. JK: Chemical validation by mass-spectrometry, checking the manuscript. PR: Chemical validation by mass-spectrometry, checking the manuscript. HK: Chemical validation by mass-spectrometry, checking the manuscript. AR: PI, acquisition of funding, checking the manuscript. MO: PI, study conceptualization, acquisition of funding and checking manuscript. AS: PI, study conceptualization, acquisition of funding and checking manuscript. PD: main PI, study conceptualization, acquisition of funding, writing and checking the manuscript. All authors contributed to the article and approved the submitted version.

## Funding

Swedish Research Council for Sustainable Development FORMAS (Pandora 2018-02280); The Estonian Research Council (grants PRG454 and PRG1076); Horizon 2020 innovation grant (ERIN, grant no. EU952516); Enterprise Estonia (grant no EU48695); MSCA-RISE-2020 project TRENDO (grant no 101008193), and Jane and Aatos Erkko Foundation (2015 “Keeping eggs healthy”).

## Acknowledgments

We thank Prof Asko Uri for the provided kinase probes, and Prof Christian Lindh for providing us the MEHP and BPF stock solutions.

## Conflict of interest

The authors declare that the research was conducted in the absence of any commercial or financial relationships that could be construed as a potential conflict of interest.

## Publisher’s note

All claims expressed in this article are solely those of the authors and do not necessarily represent those of their affiliated organizations, or those of the publisher, the editors and the reviewers. Any product that may be evaluated in this article, or claim that may be made by its manufacturer, is not guaranteed or endorsed by the publisher.
