# Supplementary material for: Endocrine disrupting chemicals interfere with decidualization of human primary endometrial stromal cells in vitro
Source: Front Endocrinol (Lausanne). 2022 Aug 19;13:903505. doi: 10.3389/fendo.2022.903505 (PMC9437351; doi:10.3389/fendo.2022.903505)
Supplement: Supplementary file 1 [file DataSheet_1.pdf]

Endocrine disrupting chemicals interfere with decidualization of human primary endometrial stromal cells *in vitro*

Darja Lavogina, Nadja Visser, Külli Samuel, Eva Davey, Richelle Duque Björvang, Jasmin Hassan, Jani Koponen, Panu Rantakokko, Hannu Kiviranta, Ago Rinken, Matts Olovsson, Andres Salumets, Pauliina Damdimopoulou

Supplementary materials

Figure S1. Characterization of selectivity of the inhibitors used for the kinase activity assay page 2

Figure S2. Characterization of isolated stromal cells page 3

Figure S3. Viability following EDC exposures page 4

Figure S4. EDCs cause reduction of IGFBP1 secretion page 5

Figure S5. Inter-woman variability page 6

Figure S6. Effect of EDCs on ELISA and Bradford assay components page 7

Table S1. MS validation of the EDC stocks page 8

Table S2. Setup of the recombinant kinase assay page 9

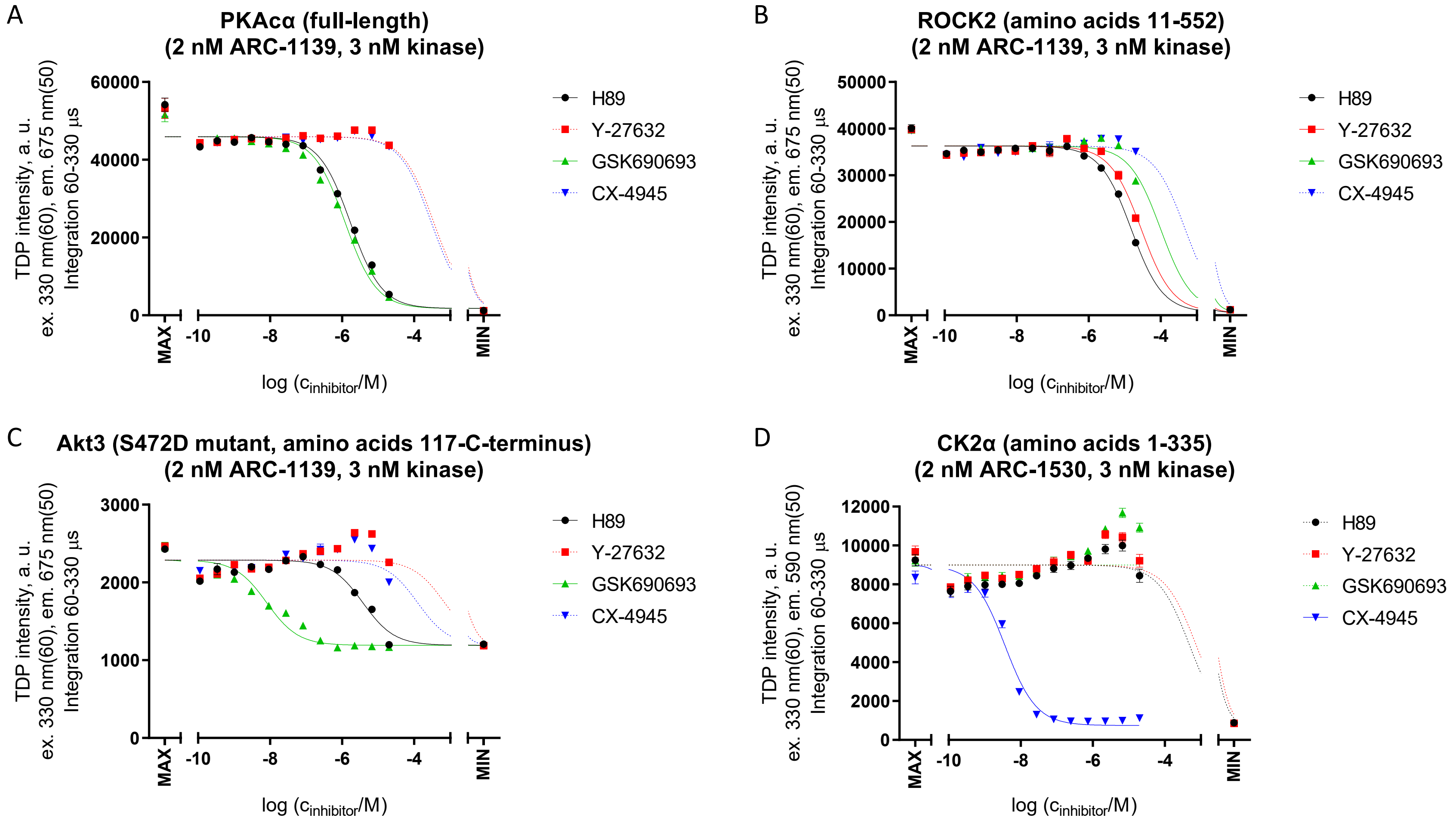

Figure S1. Characterization of selectivity of the inhibitors used for the kinase activity assay. *Recombinant protein kinases A) full-length PKAcα; B) His6-ROCK2 (amino acids 11-552); C) His6-Akt3 (S472D mutant, amino acids 117-end); D) CK2α (amino acids 1-335) were used. The graph shows the dose-response data (performed in duplicates); MAX indicates the signal of probe in the presence of protein kinase and in the absence of displacing compound, whereas MIN indicates the signal of non-bound probe. Solid lines show the fit of curves to the logarithmic dose-response model; dashed lines indicate fit that did not converge. Note that in case of H89, the IC50 value in assay with ROCK2 is almost an order of magnitude higher than in assay with PKAc (15 µM vs 1.7 µM), and in assay with Akt3, the signal measurement window is over an order of magnitude lower than in assay with PKAc (maximal signal of 2500 vs 50000).*

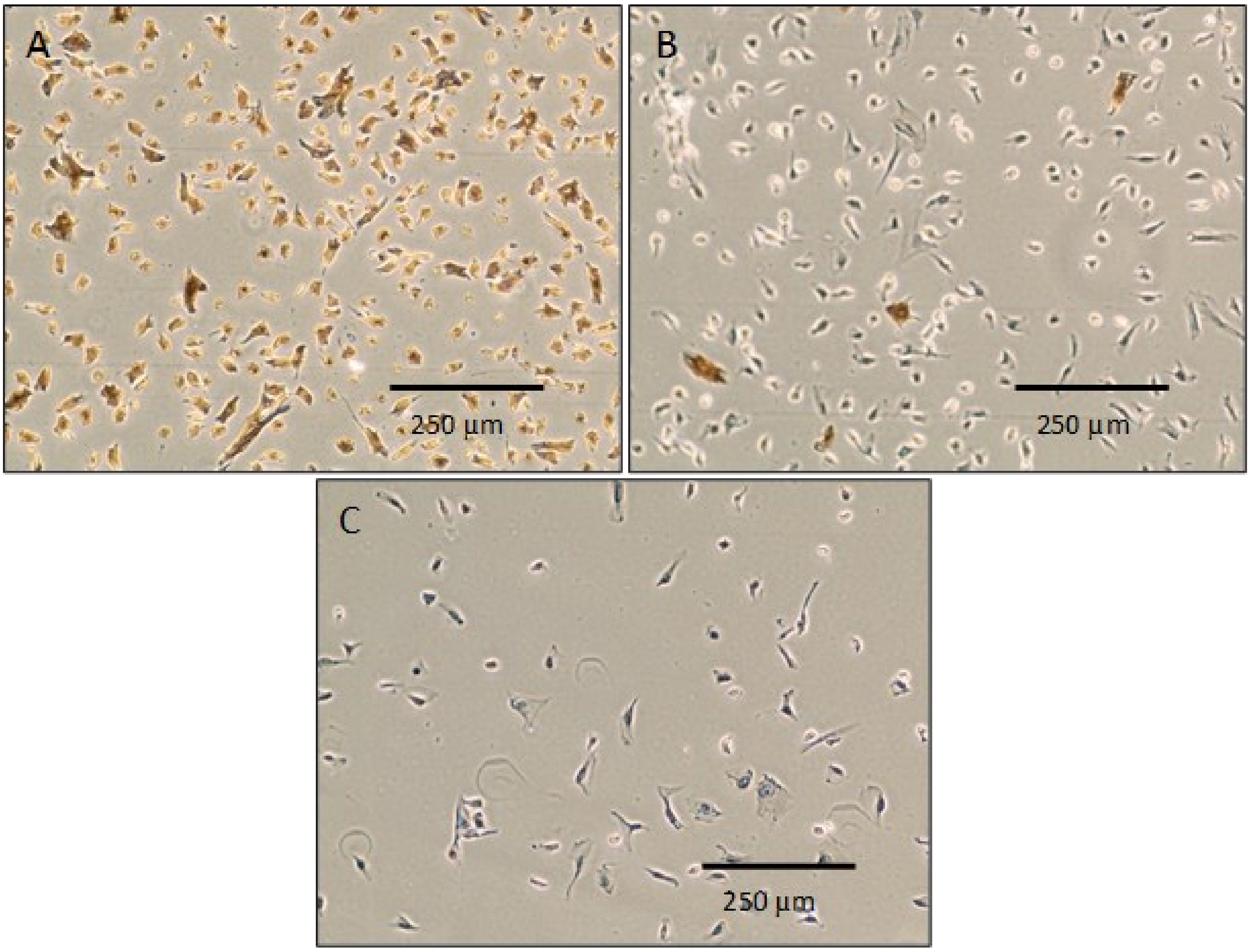

Figure S2. Characterization of isolated stromal cells. *Cells were immunostained for stromal and epithelial cell surface markers: A) vimentin for stromal cells, B) cytokeratin 8/18 for epithelial cells and C) mouse IgG as a negative control. Brown staining indicates the presence of cell surface markers. Scale bar size: 250 μm*

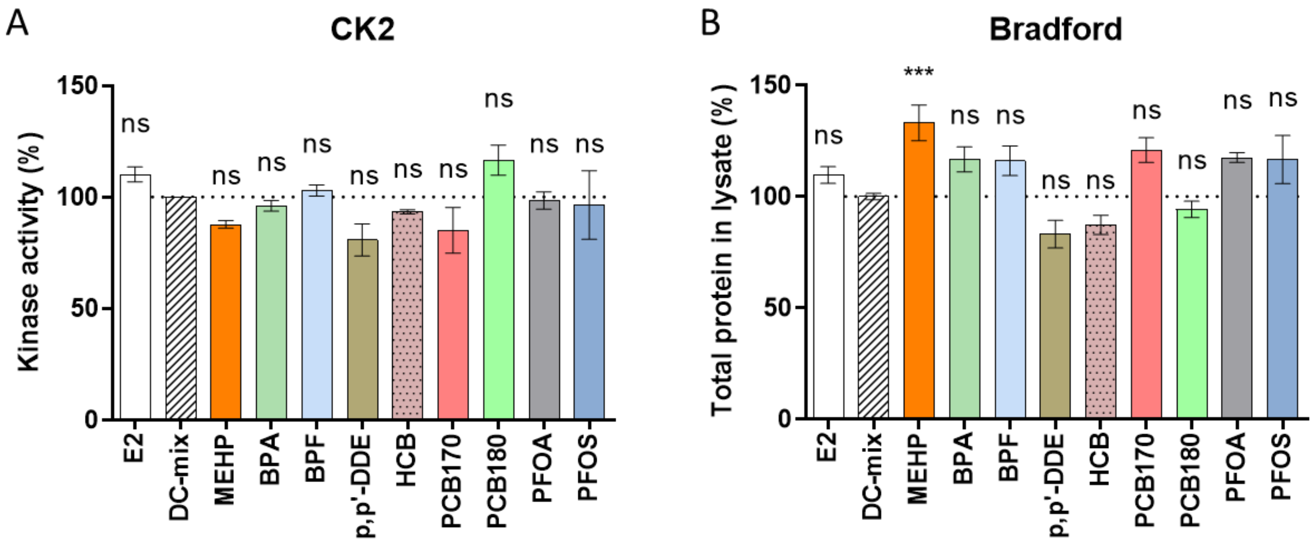

Figure S3. Viability following EDC exposures. *eSCs from two women were exposed to the decidualization-inducing mixture (DC-mix) in the absence or presence of 9 different EDCs (1 µM) for 9 days. 10 nM E2 was used as negative control. A) Viability measured by protein kinase CK2 activity in lysates (N=2) and B) total protein content in lysates measured by Bradford assay as percentage of DC-mix (N=2). Altogether 10 independent assays were carried out (n=10). Values are expressed as mean percentage of DC-mix alone ± SEM. Significance is calculated by one-way ANOVA with Dunnett test for multiple comparisons (95% CI) relative to DC-mix and indicated as ***P≤0.001 and ns, not significant. The dotted line corresponds to 100%.*

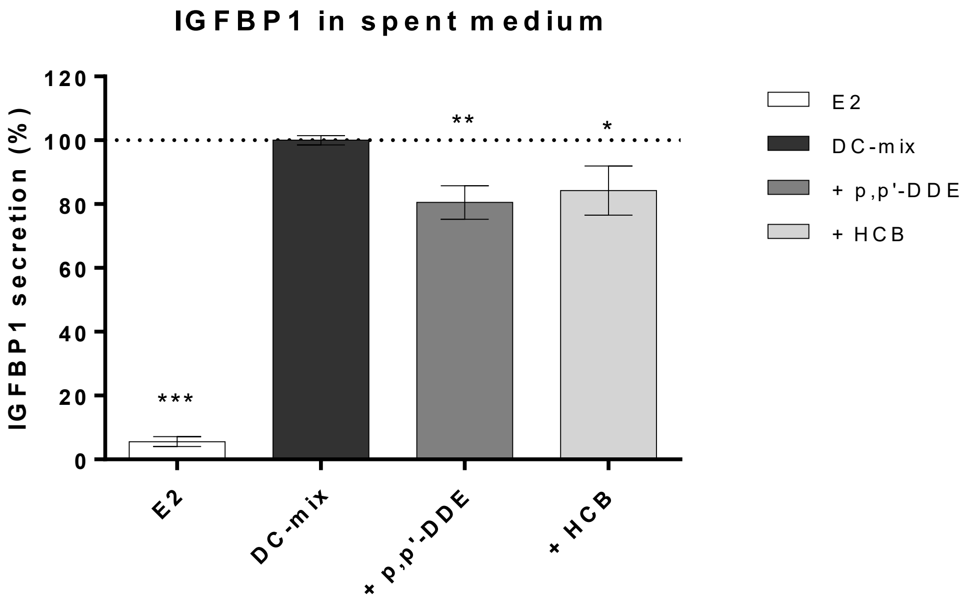

Figure S4. EDCs cause reduction of IGFBP1 secretion. *eSCs from three women were exposed to the decidualization-inducing mixture (DC-mix) in the absence or presence of 1 µM p,p'-DDE or HCB for 9 days. 10 nM E2 was used as negative control. Secreted IGFBP1 (medium aliquots collected following last 72 h treatment at the end of in vitro decidualization) was calculated as percentage of DC-mix. Values are expressed as mean percentage of DC-mix alone ± SEM (N = 2, n = 10). Significance is calculated by one-way ANOVA with Dunnett test for multiple comparisons (95% CI) relative to DC-mix and indicated as *P<0.05, **P<0.01 and ***P<0.001. The dotted line corresponds to 100%.*

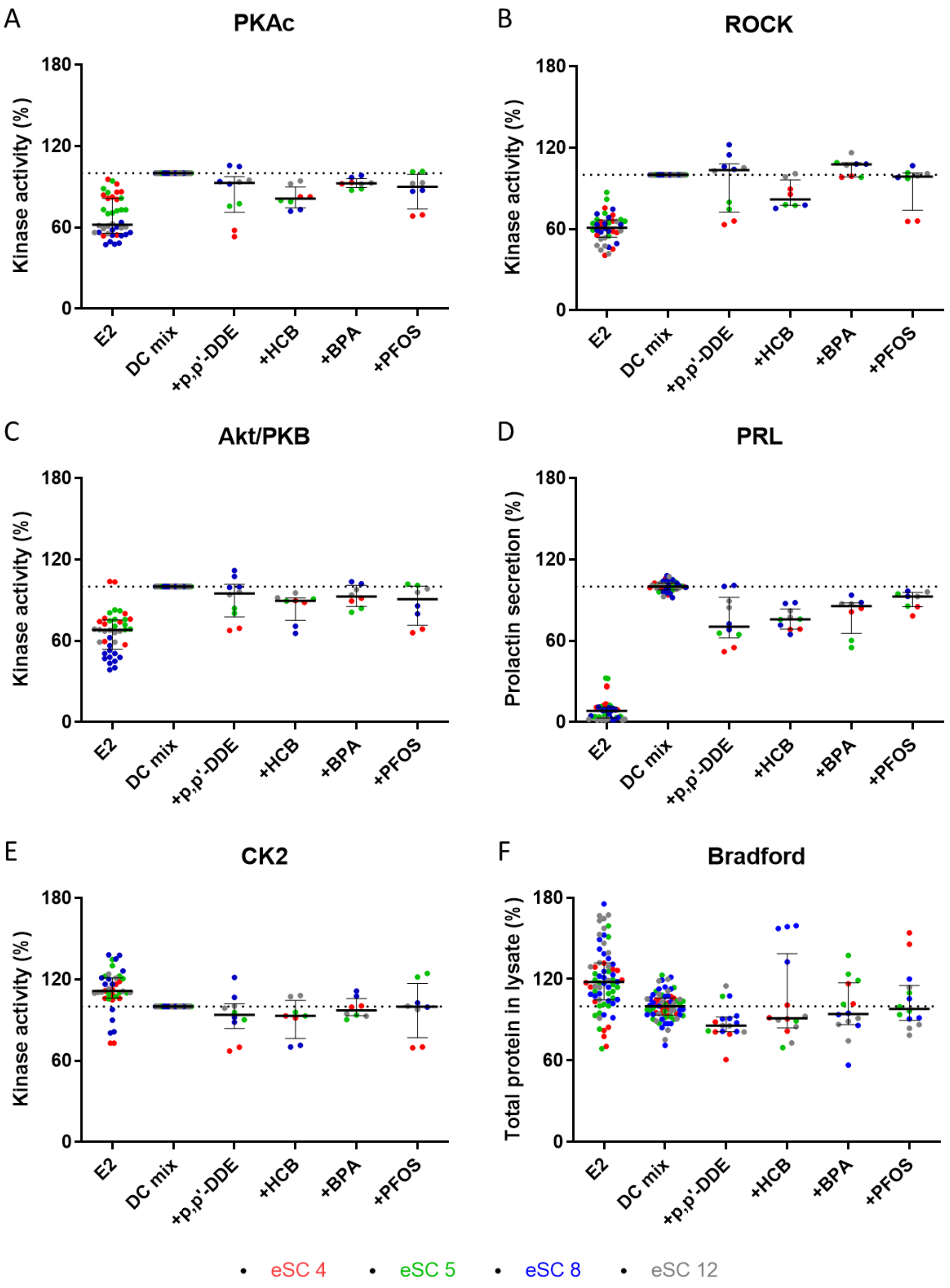

Figure S5. Inter-woman variability. *eSCs from four women were exposed to the decidualization-inducing mixture (DC-mix) in the absence or presence of 1 µM p,p'-DDE, HCB, BPA, or PFOS for 9 days. 10 nM E2 was used as negative control. Protein kinase activity in lysates was measured as percentage of DC-mix (100%) for A) PKAc, B) ROCK, C) Akt/PKB. Secreted PRL (D, medium aliquots collected following last 72 h treatment at the end of in vitro decidualization) was calculated as percentage of DC-mix. The impact of the exposures on viability of the eSCs in culture during decidualization assays was assessed by the activity of the viability marker kinase CK2 (E) as well as evaluating protein concentrations in lysates at the end of the assay (n=4-6) (F). eSCs from different women are indicated in different colors: red eSC4; green eSC5; blue eSC8; grey eSC12 and each independent experiment was carried out in duplicates. Negative and positive controls were pooled for the protein kinases (n=4-5), PRL secretion (n=4-5) and Bradford assay (n=20). The thick line represents the median for each exposure and the whiskers show interquartile range. The dotted line corresponds to 100%.*

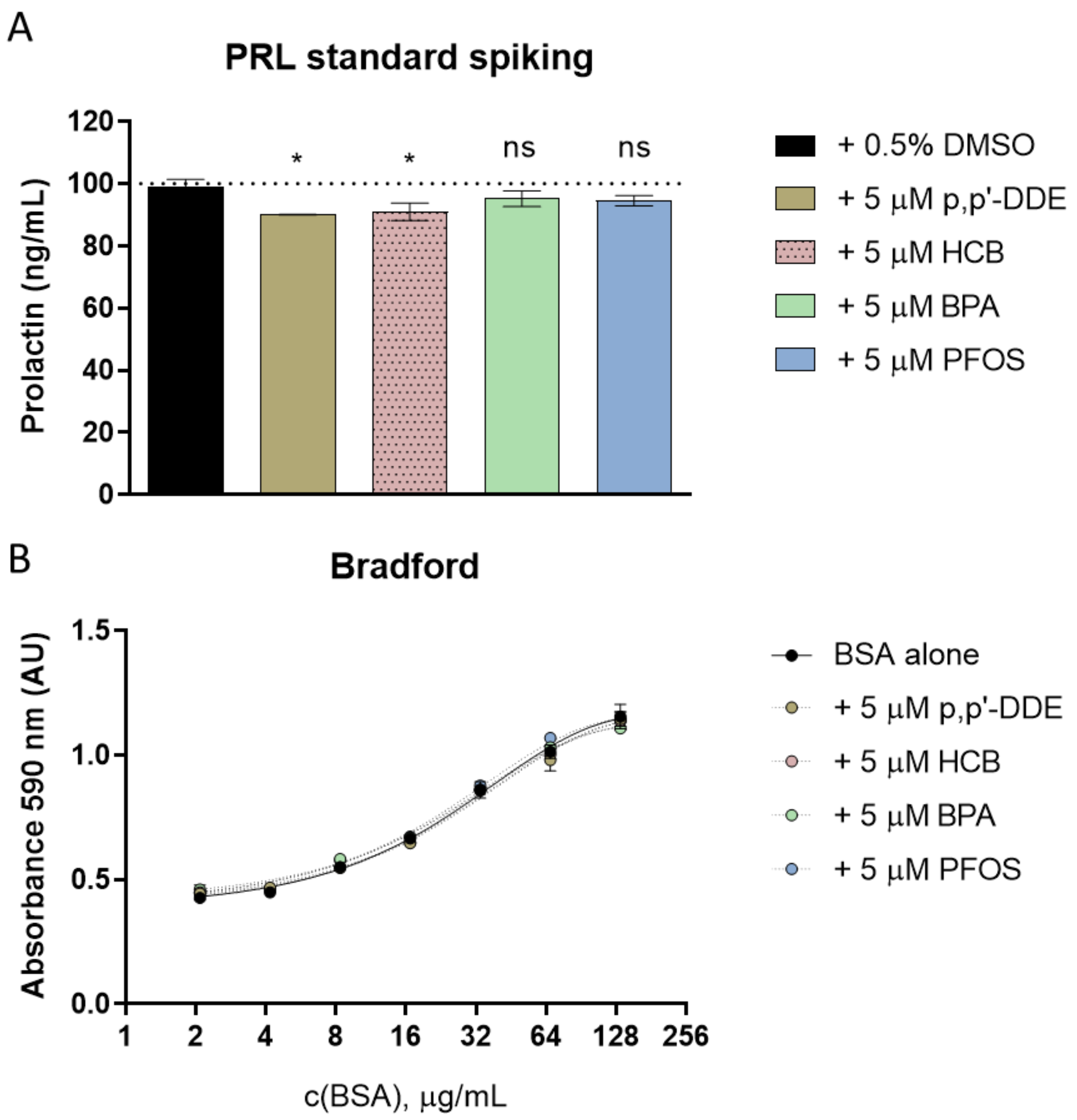

Figure S6. Effect of EDCs on ELISA and Bradford assay components. *(A) EDCs or DMSO were spiked to the 100 ng/mL PRL assay standard during one experiment in duplicates; the dotted line corresponds to 100 ng/mL standard without any spiking. Significance is indicated as *P < 0.05 and ns, not significant. (B) EDCs were spiked to the BSA dilution curve. The experiment was performed once in duplicates.*

Table S1. MS validation of the EDC stocks

**EDC**	**Mw g/mol**	**Nominal* μg/L**	**Measured μg/L**	**Measured μM**	**Recovery %**
**MEHP**	278	556	616	2.216	110.8
**BPA**	228	456	550	2.412	120.6
**BPF**	200	400	520	2.600	130.0
**p,p'-DDE**	318	636	511	1.607	80.3
**HCB**	285	570	494.25	1.734	86.7
**PCB170**	395	790	156.25	0.396	19.8
**PCB180**	395	790	351.5	0.890	44.5
**PFOA**	414	828	920	2.222	111.1
**PFOS**	500	1000	940	1.880	94.0

*The nominal concentrations of EDC stock aliquots submitted to the MS facility were 2 μM.

Table S2. Setup of the recombinant kinase assay

Protein kinase *	Probe*	Control inhibitor *	Measurement conditions
**PKAcα (1.3 nM or 3 nM)**	ARC-1139 (2 nM) or ARC-583 (2 nM)	H89 (1 μM)	TDP, ex 337 nm, em 675 nm; or FA, ex 540 nm, em 590 nm
**ROCK2 (3 nM)**	ARC-583 (2 nM)	Y-27632 (1 μM)	FA, ex 540 nm, em 590 nm
**Akt3 (3 nM)**	ARC-1139 (2 nM)	GSK-690693 (1 μM)	TDP, ex 337 nm, em 675 nm
**CK2α (1.7 nM or 3 nM)**	ARC-1530 (2 nM)	CX-4945 (1 μM)	TDP, ex 337 nm, em 590 nm

*Final total concentrations in the assay mixture are indicated in brackets. FA stands for fluorescence anisotropy and TDP for time-delayed photoluminescence.
